# Understanding the Role of Social Interactions in the Development of an Extracurricular University Volunteer Activity in a Developing Country

**DOI:** 10.3390/ijerph17124422

**Published:** 2020-06-19

**Authors:** Ariane Díaz-Iso, Almudena Eizaguirre, Ana García-Olalla

**Affiliations:** 1 Department of Innovation and Educational Organization, University of Deusto, Avenida de las Universidades, 24, 48007 Bilbao, Spain; ana.garciaolalla@deusto.es; 2 Management Department, University of Deusto, Hermanos Aguirre, 2, 48014 Bilbao, Spain; almudena.eizaguirre@deusto.es

**Keywords:** extracurricular activities, volunteering, social interactions, well-being, attitude toward social transformation, adaptation to university life, professional development, academic performance

## Abstract

The relevance of participating in structured extracurricular activities (ECA from here onwords) in higher education is increasing. Involvement in these activities helps students develop personal and social skills that positively affect academic and professional training, well-being, and the development of attitudes toward community involvement. This study analyzes the role of social interactions in the perceived benefits of students, who have participated in an ECA in a developing country. Moreover, this research aspires to explore whether students perceive that these interactions positively impact academic training, professional development, and adjustment to the university context, psychological well-being, and development of community involvement attitudes. As a result, 46 in-depth interviews were conducted with 23 students who participated in the experience. 23 in-depth interviews were conducted before the experience and another 23 after it. Data analysis was carried out using the IRaMuTeQ software to conduct a descending hierarchical classification (DHC). This study highlights the value of social interactions in an ECA to increase the motivation of students to improve academic and professional performance, to build shared knowledge with others, and to develop personal and social skills that contribute to the integral development of participants. ECAs help students reflect on their actions and privileges and develop positive attitudes toward themselves and others. This fact is linked to the achievement of high levels of well-being that allow the enhancement of the students’ self-esteem. Finally, this experience has allowed volunteers to become aware of other sociocultural realities and to reflect on the possible ways of contributing to the development of a more sustainable society.

## 1. Introduction

Recently, researchers have shown an increased interest in extracurricular activities (ECAs) in higher education. This line of research maintains that such activities are ideal for promoting spaces where students can interact and integrate with their peers; with people from other cultures, religions, and ideologies; or with teachers [1]. Concerning university ECAs, we refer, as defined by Díaz-Iso et al. [2], to those voluntary activities that complement curricular training, which takes place outside the class schedule and contribute to the students’ personal, professional and social development. These activities are classified into volunteering, sporting, cultural, spiritual and artistic activities, and student clubs.

Currently, one of the biggest challenges in higher education is to foster personal attitudes among students that will enable them to move toward more equitable, just, and sustainable societies. In this context, a growing body of literature has studied how ECAs contribute to this goal [3,4,5]. Furthermore, research in this field has indicated the impact of ECA in terms of achieving higher levels of academic performance [6], having more successful development during university life [7], improving later employment adaptation [8], and obtaining more significant social and psychological resources that aid the students’ mental well-being [9].

Due to the interactions taking place in ECAs, students are presented with the opportunity to create stronger bonds with the community. Through ECA and the emerging links, students can respond to the sustainability challenges faced by society today [5]. Currently, encouraging participation in ECAs is relevant for two main reasons: (1) because higher education institutions are called upon to promote quality experiences that enable students to acquire skills, abilities, and knowledge that will allow them to be active and responsible citizens [10] and (2) because paying attention to scientific evidence that indicates the importance of implementing effective and quality interventions, with professionals and peers, that encourage positive interactions and enable students to transform social challenges is necessary [11,12,13].

Until now, only few qualitative methods have been developed to understand the benefits of social interactions created by ECAs [1]. However, as Winstone et al. [14] point out, there is still a need to expand research on ECA in higher education to include studies with different student populations and across a broader range of socioeconomic contexts and countries. Additionally, past research neither identifies or clarifies all the functions that ECAs can perform in higher education nor analyzes how each particular type of ECA fulfills these functions. Several authors [14,15,16] also highlight the importance of recognizing students’ nuanced and individual experiences and avoiding homogenizing the students’ experience. Therefore, qualitative analyses that address these issues in greater depth and that provide a detailed picture of each students’ perceptions are crucial [17,18,19]. This type of analysis allows us to adjust the protocol to each case and make the necessary inquiry for each particular context. This would help provide a greater comprehension of how students see the value and importance of extracurricular activities and could guide universities in supporting students to benefit from that engagement.

This study explicitly analyzes an ECA performed by Spanish university students with children at risk of exclusion and people with disabilities living in a disadvantaged urban area of Tangier (Morocco). Specifically, it analyzes a short-term international volunteering activity that 23 students from a Spanish university conducted in Tangier (Morocco).

**Figure 1:**
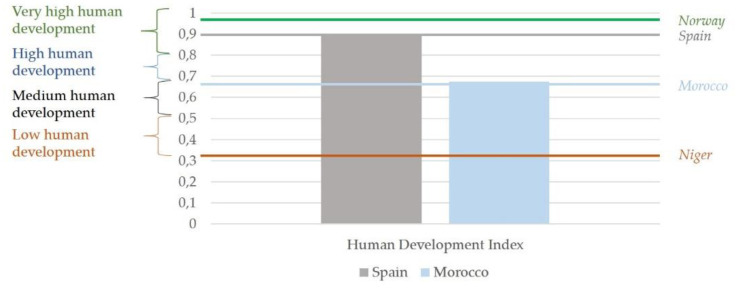
Score obtained in each country according to the Human Development Report published in 2019.

This extracurricular volunteer activity seeks to give students a group experience in a developing country, where they are in contact with another culture and learn about the reality of immigration on the other side of the border. According to the Human Development Report [20], Spain, with a Human Development Index (HDI) of 0.893, is ranked as the 25th most developed country, whereas Morocco (HDI = 0.676), where students conducted the volunteer experience, ranked 121st (see Figure 1).

This study explores the role of social interactions in the perceived benefits of students participating in an extracurricular volunteer activity in a developing country. Further, it seeks to explore whether students perceive that these interactions have a positive impact on academic training, professional development, and adaptation to the university context, psychological well-being, and development of responsible attitudes in the community. The interactions among students, interactions of students with their instructor, and interactions of students with the populations at risk of exclusion with whom they interact in the experience are the objects of this study.

## 2. Role of Social Interactions in Developing an ECA

ECAs are activities that take place in shared and diverse contexts. Through dialog and the creation of a climate of confidence among participants, students have the opportunity to share feelings and reflect on their engagement and contribution to society [5,21]. This conceptualization is based on the contributions of the sociocultural theory of human learning, which conceives learning and cognitive development as a process of joint construction that occurs in the course of interaction [22,23].

These activities conducted voluntarily allow students to assume more meaningful commitments and responsibilities with their actions in the society. Furthermore, they promote key learning processes for the development of personal and social skills that favor the socialization of students [24,25]. The development of these skills, such as active listening, negotiation, or teamwork, is also beneficial for developing attitudes that allow higher levels of academic performance [6], for a more successful university life [7], and for the improvement of job performance [8].

The contribution of ECAs to the development of social and psychological resources that help the mental well-being of students has also been analyzed [14,26,27]. An increasing number of studies have found a significant positive relationship between engagement in extracurricular activities and extraversion (i.e., sociability and positive emotionality). This extraversion is positively related to a higher psychological sense of community, greater involvement in campus activities and subjective wellbeing [14,27]. Therefore, ECAs are considered suitable for boosting confidence [19,28], sense of belonging [29], self-esteem [30], and positive self-concept [31], as well as developing emotional skills that enable a more positive attitude toward self and others [1].

Students can also share reflections, feelings, knowledge, and experiences with others through conversations with people from different social and cultural contexts. These interactions promote the development of critical thinking and reflection on the values of students. This exchange of experiences and thoughts allows, on the one hand, to produce reflective processes that help students understand themselves, their needs and problems, and their strengths and limitations [21,32] and, on the other hand, to assimilate, exchange and analyze divergent perspectives and understand the views of other people [33,34].

Participation in ECAs allows students to develop skills and attitudes that help them to understand other sociocultural realities, identify social problems, and participate in peaceful conflict resolutions [35]. Hence, it is argued that they promote lifelong learning, active citizenship and a sense of social justice, which enables students to assume more meaningful roles in society and face daily tasks with a more positive state of mind [4,36].

Moreover, the construction of meaning is carried out jointly in interaction with other people. These interactions are an essential step to make the learning process sensible, to maximize the learning results of the experience of students, and to respond to the challenge of achieving the integral formation of students [37].

## 3. Materials and Methods

### 3.1. Context

This study analyzes a short-term international volunteering activity that 23 students from a Spanish university conducted in Tangier (Morocco). Of the 23 students, 13 participated in the volunteer activity in January 2019 and 10 participated in June 2019.

During ECA, students participate in three different social projects. (a) The Dar Al Baraka project of Casa Nazaret, a foster home that assists 10 people with intellectual or physical disabilities—these people do not have family assistance or resources to live on their own. (b) The Dar Tika project is a shelter that incorporates the medical, psychological, and social monitoring of girls between 11 and 14 years old. They are girls at serious risk of social exclusion, without family, who have ended up living on the street or have suffered abuse and aggression. (c) The Father Lerchundi project, a day center attended by children aged 6–16, enrolled in Moroccan public schools, during the hours when they do not have school. They come to this center to eat, shower, play, and receive school support. In this way, this center prevents them from falling into the nets of evildoers or developing bad habits on the street.

The students worked mainly on the Dar Al Baraka project at Nazareth House. They helped in the day-to-day activities of the people with disabilities living at the center. The accompaniment aimed to help them establish a daily routine to be more autonomous in everyday activities, such as eating, bathing, or dressing. Further, every morning, a small group of students had the opportunity to give school support and accompaniment in their free time to the children of the Father Lerchundi project. One morning, all the participants could also visit the Dar Tika project and get to know the main lines of action of the project. Finally, some participants went to the border of Ceuta, where they had the opportunity to learn about the harsh reality of people who want to emigrate to improve their living conditions. The volunteers also shared the housework, and at the end of each day, the students did a guided reflection led by their instructor. In these meetings, the students, through interaction with their peers and mutual help, shared different knowledge, reflections, and sensations experienced during the day to learn from one another.

### 3.2. Sample

As stated earlier, 23 students (19 women and four men) enrolled in a Spanish university participated in this volunteer experience. The participants aged from 18 to 24 years. Table 1 shows the distribution by course, degree, and previous experience in extracurricular volunteer activities. The 23 students were divided into two groups. Group 1 (*n =* 13) participated in the ECA in January 2019 and Group 2 (*n =* 10) in June 2019.

The students pursue their undergraduate studies in a social initiative non-profit university. The socioeconomic background of these students is mostly middle-class and, to a lesser extent, upper-middle-class. Thus, these students must pay moderate tuition fees. In addition, some students obtain financial aid from university scholarships and other external financial institutions.

### 3.3. Research Tool: In-Depth Open Interviews

As some research has asserted, the majority of recent studies have measured students’ perceptions of ECAs’ impact on their integral development through quantitative research techniques, such as questionnaires [38,39]. However, in this study, to allow students to deepen the experience and reflect on their interactions from the activity, semi-structured interviews were conducted. More specifically, for this research work, 46 semi-structured interviews were conducted with the 23 participants before and after volunteering experience in Tangier (Morocco).

Through the interviews, the participants were able to explain their feelings or perceptions generated regarding the social interactions during the experience to highlight the themes or issues that were most relevant to them [40,41]. In-depth interviews are considered an ideal data collection technique for creating links between the researcher and interviewee. Moreover, exploring the participants’ experiences and collecting the various ideas, knowledge, and impressions that the interviewees have about it are useful [42,43].

The question script of the semi-structured interview is based on a systematic review of the concept of ECAs in higher education. As a result of the review of 50 studies on the subject, identifying the five main functions provided by ECAs was possible: they favor the employability and professional development, academic performance, well-being, university experience, and social participation and transformation of students.

Our goal was to understand more comprehensively the role of social interactions in the perceived benefits of students participating in the experience, specifically whether these interactions have a positive impact on the academic training, professional development, adaptation to the university context, psychological well-being, and the development of community involvement attitudes of the students. Hence, five questions were formulated (Table 2). The participants were also asked questions about their age, course, grade, and previous volunteer experience. The design of the question script was approached considering the consolidated criteria for reporting qualitative studies (COREQ criteria). Finally, to guarantee the validity of the question script, it was subjected to an assessment by three experts in didactics and university education [43].

Moreover, to guarantee the validity of the interview schedule, it was subjected to an assessment by three experts. Finally, the participants were asked questions about their age, course, and grade.

### 3.4. Data Collection Procedure

Before the students participated in the ECA, the researchers contacted them to explain the purpose of this study and ask for collaboration. We met the students on campus before they participated in the experience. All the participants volunteered for the interview without any extra incentive. After agreeing to participate in the study, they were asked to sign an informed consent form. Each semi-structured preinterview took around 25 min, while the postinterviews took around 45 min.

Group 1 interviews were conducted between January and February 2019 and Group 2 interviews in June 2019. All of them were conducted in a safe and trusting environment to access the personal experiences of the students [44]. The interviews were audio recorded with the consent of the students. The interviewer was the same person (one of the study researchers) in all interviews, taking place in a university classroom. The interviews were conducted as natural conversations that helped us access the students’ experiences. The interview guide contained approximately five questions. Through the interview, we encouraged students to share more of the details and insights of their experiences.

### 3.5. Data Analysis

The data analysis has been conducted using the IRaMuTeQ software to carry out the analysis of the information and efficient treatment of the data obtained. The IRaMuTeQ software (R interface for the multidimensional analysis of texts and questionnaires) was created by Pierre Ratinaud to meet the needs of social and human science research, wherein the linguistic material analysis of the different types of documents was conducted. This open-source software is very convenient for working with large amounts of texts because it identifies patterns that would otherwise be highly difficult to detect [45,46].

This software is based on the R software and allows distinct types of analysis. Among them, it can carry out a hierarchical descending cluster analysis [47,48,49] to analyze the lexical similarities and differences in the texts to be later able to recognize the repetitive patterns of language [50]. The DHC method classifies text segments according to the words or vocabulary it is composed of. Thus, the software algorithm analyzes the texts, identifies the most frequent words, and gathers the lexical worlds (set of words that make up a discourse fragment) expressed by the interviewees [51]. This analysis aims to obtain classes with a similar vocabulary that, at the same time, differentiates them from the rest of the classes. The chi-square test (x2) is used to show the associative force between the words and their respective class. Moreover, through a factorial correspondence analysis, knowing the different variables associated with each class and retrieving the most characteristic text segments related to each of them, which allows for a qualitative analysis of the data, are possible [52].

Therefore, the analysis of the information was conducted in divergent stages: the codification of the interview transcriptions, the descending hierarchical classification carried out for data processing, and the interpretation of the classes. If we compare the Reinert method with the classical content analysis, we could see that while the classical content analysis carries out the interpretative process when building the coding categories, the IRaMuTeQ software moves the interpretative process to the later moment of performing the statistical analysis [53]. Therefore, for the interpretation of the data, taking the results obtained in the systematic review [2], as well as the triangulation of researchers, as a reference has been essential. As Yin [54] stated, in this study, this triangulation is justified by the opportunity it offers to compare, contrast, neutralize, and highlight the different biases of the disciplines. Thus, the researchers conducted the data analysis independently, and subsequently compared these analyses. The reported results of the study were the product of the research work carried out by the three researchers. Their different backgrounds (from the field of management, education, and innovation) have allowed the object of study to be analyzed by experts in distinct areas and to understand the phenomenon through multiple facets.

## 4. Results

**Figure 2:**
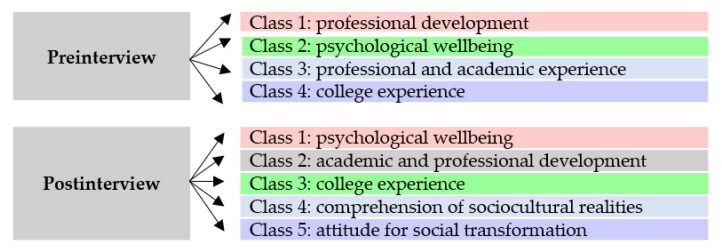
Distribution of the classes in pre and postinterviews.

The complete corpus of the interviews includes 51,586 words (25,317 words of the presubcorpus and 26,269 words of the postsubcorpus). The DHC analysis of the preinterviews classified the subcorpus into four classes, while the subcorpus of the postinterviews was classified into five classes (Figure 2).

### 4.1. Pre-Test Results

**Figure 3:**
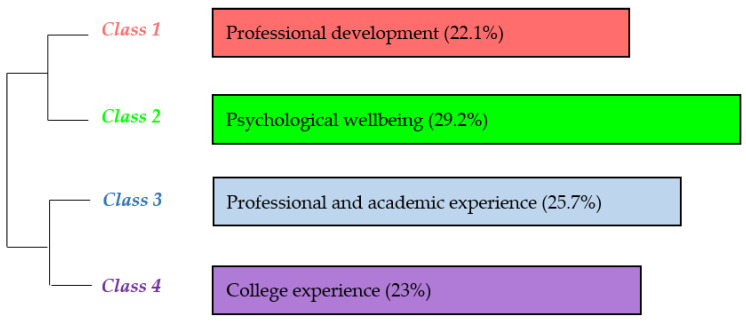
Dendrogram of the preinterview classes.

Figure 3 depicts that the four main classes (or lexical worlds) identified in the interviews conducted before the experience refer to professional development (class 1, 22.1% presence in the corpus); psychological well-being (class 2, 29.2% presence in the corpus); professional and academic experience (class 3, 25.7% presence in the corpus); and university experience (class 4, 23% presence in the corpus). A detailed description of each class can be found below.

Class 1: Professional Development

The first class corresponds significantly to the categories of social transformation (x^2^ = 16.94, *p* < 0.0001) and professional development (x^2^ = 12.38, *p* < 0.00043).

Participants in this class expect that this experience can help them to develop (x^2^ = 10.5) relevant skills (x^2^ = 6.3) that can contribute to better professional performance. “Public speaking and knowing how to respond to complicated situations are important for my professional career” (participant 3; score 157.55). At the same time, they believe that interactions with people who live in other sociocultural realities can be useful to understand (x^2^ = 9.09) and know (x^2^ = 11.99) different situations (x^2^ = 12.51) or contexts in which they may find themselves in their future work environment. “When I become a teacher, for example, if I work with Muslim students, it will help me to understand their religion better” (participant 7; score 84.57). In addition, working with people from these two (x^2^ = 6.52) groups (x^2^ = 7.4), children at risk of exclusion and people with disabilities can be positive for future job opportunities (x^2^ = 46.11). “Working with children and people with disabilities are two sectors that give me much respect; thus, putting that on the curriculum is very much appreciated. I know this because I did an internship at Cruz Roja, and they told me a lot” (participant 6; score 178.07). In the same way, they reveal that it can help them obtain an idea (x^2^ = 30.08) or realize (x^2^ = 16.34) what they want to do when they finish their university studies. “Maybe I go to Morocco, and I realize that I am not passionate about working with foreigners, or maybe I suddenly think that women are not the group I want to work with” (participant 13; score 108.80).

Class 2: Psychological Well-Being

The second class is significantly related to the categories of well-being (x^2^ = 106.15, *p* < 0.0001) and social transformation (x^2^ = 10.34, *p* < 0.00130).

Interviewees relate their experience to the development of attitudes that can help them (x^2^ = 51.45) feel (x^2^ = 68.69) better about themselves (x^2^ = 37.1). “Although you will logically have some hard times during the experience, I think it makes you feel better, more self-reliant, cooperative, and involved. It makes you feel good” (participant 15; score 179.64). Students say that living this experience can enhance the reflection and awareness of the excessive privileges they have. This can sometimes make them feel selfish (x^2^ = 17.18) or affect (x^2^ = 21.0) negatively. “Maybe it does help me feel more conscious and consistent with my ideas. It will not help me to consider myself a better person because in the end, the reality I am living is completely different. My real life is full of comforts and with certain needs covered and even so, many times, I complain and claim about unnecessary things” (participant 9; score 166.84)

Class 3: Professional and Academic Experience

The third class is closely linked to the categories of professional development (x^2^ = 16.94, *p* < 0.0001) and academic performance (x^2^ = 16.94, *p* < 0.0001).

Students believe that volunteering (x^2^ = 45.77) can provide them with a plus (x^2^ = 25.0) of professional (x^2^= 9.98) experience (x^2^ = 10.31) that will facilitate (x^2^ = 8.73) the search of employment (x^2^ = 39.78) in the future (x^2^ = 43.09). “In the end, volunteering for me is also an experience; it is not a job, but it is a professional experience for a future” (participant 20; score: 158.26). At the same time, they report that this practical experience (x^2^ = 41.51) can complement the theoretical knowledge (x^2^ = 13.22) of the degree (x^2^ = 25.72) they are studying.” With this type of volunteer experience, you acquire knowledge that you have worked on theoretically, but until you put it into practice, you do not internalize it” (participant 22; score: 153.36). In this class, it is also stated that the experience can be useful to give a social (x^2^ = 45.89) sense to the formation they are taking. “All university students can learn and become more aware of the problems of others and the situation in which other people live. Sometimes this concern is very present in studies such as Social Education, but it is important in all types of studies” (participant 23; score: 122.72).

Class 4: College Experience

The fourth class is significantly associated with the category of the college experience (x^2^ = 111.62, *p* < 0.0001).

Students value that the university has offered them (x^2^ = 12.39) the opportunity (x^2^ = 9.45) to be part (x^2^ = 19.45) of this experience, and this has encouraged them to change the image or concept they had about the university. “For some people, college is a continuation of school. You wake up, you study, you go home to sleep, and the next day you do the same routine again. But I think college gives people many opportunities to do other kinds of activities and meet different people” (participant 13; score: 169.99). Further, students expect that participation in ECAs will enrich their experience at the university (x^2^ = 71.39). “It can be an enriching experience outside of the academic formation. It is a plus to add to your university experience” (participant 15; score: 127.16). Many of the students relate this enrichment to the opportunity to interact with their peers and to be part (x^2^ = 19.45) of the university. “I think it helps to connect with different students from different careers and to create a spirit of doing things within the university” (participant 4; score: 119.33). Other participants see this volunteering as an opportunity for personal development (x^2^ = 4.83). “During the university years you not only have to acquire professional training, but I think you also have to develop as a person. For this, you have to do more activities apart from going to class or taking exams” (participant 18; score: 138.77).

**Figure 4:**
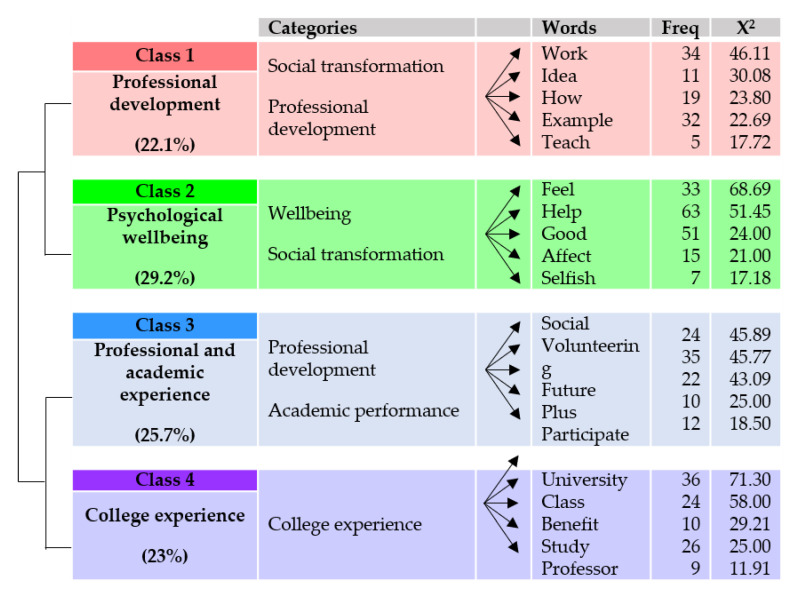
Distribution of classes and their respective units of meaning (preinterviews).

Figure 4 shows the representation of the main classes and subclasses resulting from the IRaMuTeQ descending hierarchical classification analysis of the corpus of pre-interviews.

### 4.2. Post-Test Results

**Figure 5:**
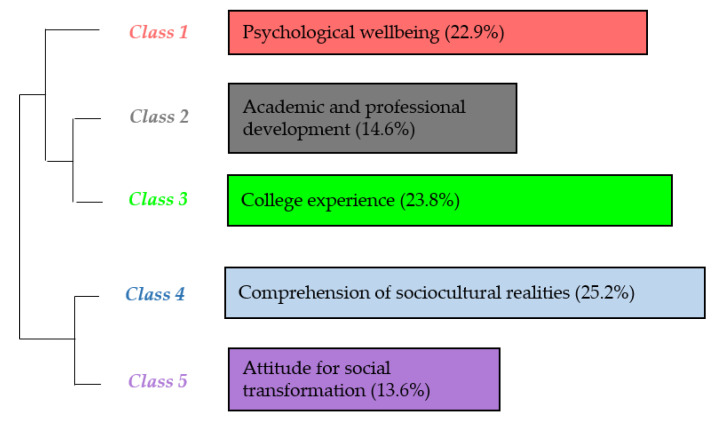
Dendrogram of the postinterview classes.

Figure 5 illustrates that the five main classes (or lexical worlds) identified in the postinterviews refer to psychological well-being (class 1, 22.9% presence in the corpus); academic and professional development (class 2, 14.6% presence in the corpus); college experience (class 3, 23.8% presence in the corpus); the comprehension of sociocultural realities (class 4, 25.2% presence in the corpus); and attitude for social transformation (class 5, 13.6% presence in the corpus).

Class 1: Psychological Well-Being

The first class is associated with the category of psychological well-being (x^2^ = 4.11, *p* < 0.04269). It includes the reflections of students on how they feel (x^2^ = 30.87) after participating in the experience. Some people highlight that interacting with populations at risk of exclusion has made them feel selfish (x^2^ = 37.83) because they have received more than what they have given. “I have the feeling that they have helped me more than I have been able to offer them” (participant 10; score: 115.75). Other participants highlighted the positive attitudes (x^2^ = 15.81) they have developed toward themselves and society: “I already consider myself a critical person, but I believe this experience has helped me to reaffirm my attitude toward society or myself” (participant 9; score 102.15).

Class 2: Academic and Professional Development.

The second class is closely related to the categories of professional development (x^2^ = 21.7, *p* < 0.0001) and academic performance (x^2^ = 4.32, *p* < 0.03776).

In this class, ideas are gathered regarding how participating in projects (x^2^ = 10.26) with children or people with functional diversity (x^2^ = 52.37) has affected their professional and academic (x^2^ = 4.55) development. Regarding the professional field, some participants believe that it has served (x^2^ = 30.34) them to open new professional doors. “I never would have thought that I would feel comfortable working with people with functional diversity. It is as if a door for the future had been opened for me” (participant 10; score: 213.46). Regarding the academic field, students perceive that it has helped them to understand better the concepts that are worked on in the different subjects (x^2^ = 17.34) of the degree (x^2^ = 20.49). “In the academic sense, it will help me better understand the harsh reality that many immigrants experience, the need to empower women and to meet the basic needs of children …. The experience somehow awakens something in you, it is no longer just listening to it, but you have lived it in the first person. The fact of knowing children who are living injustice makes you interiorize the injustice more, and if you interiorize it, you act more and more effectively” (participant 11; score 74.26).

Class 3: College Experience

The third class corresponds significantly to the categories of university experience (x^2^ = 47.35, *p* < 0.0001) and academic performance (x^2^ = 15.04, *p* 0.00010).

This extracurricular activity has helped them to enrich (x^2^ = 8.76) their experience at the university (x^2^ = 55.48) and to value the opportunities (x^2^ = 4.41) that the institution (x^2^ = 27.03) offers them to interact with new people. “In the end, I have a more positive point of view towards the university. For example, it gives you options to do different things, apart from academic training, it offers you possibilities to develop as a person” (participant 14; score: 129.31).

Thus, in this class, the participants point out that this experience, first of all, has helped them to interact with people living in different (x^2^ = 19.55) cultural (x^2^ = 4.41) contexts and to put into practice (x^2^ = 19.36) some personal (x^2^ = 12.37) skills (x^2^ = 16.11). “You have to know people and understand how they deal with the situations they’re in. But you do not study that, you work at it by experimenting, by playing. That is why these experiences seem essential to me” (participant 18; score: 214.04). Second, they highlight that this experience has also allowed them to meet (x^2^ = 62.91) students from other degrees (x^2^ = 5.96). “I’ve met many people who are very similar to me in their way of being, and we’ve connected a lot among the people in the group. I believe that after this intense experience, we will be able to continue to meet and join together here at the university” (participant 16; score: 177.03). Finally, living the experience with students from other degrees has helped the volunteers to connect (x^2^ = 16.11) and get to know different fields (x^2^ = 8.02) and activities (x^2^ = 8.76) that they did not understand. “The girl who studies Modern Languages has told us about language projects that I didn’t know about, so you feel like signing up for other workshops” (participant 11; score: 111.35).

Class 4: Comprehension of Other Sociocultural Realities

This fourth class is closely related to the categories of social transformation (x^2^ = 29.2, *p* < 0.0001) and well-being (x^2^ = 11.83, *p* < 0.00058).

The participants, in this class, reflect on the different realities experienced by the people with whom they have been involved in the volunteering. This way, they highlight that the present experience helped them understand the way others live, concretely, the people they had the chance to work within the Casa Lertxundi (x^2^ = 17.34) project, the children (x^2^ = 52.12) and families (x^2^ = 16.58) of the school (x^2^ = 10.89), and the children who lived on the street (x^2^ = 45.41). “I remember a girl who spent her breaks studying and learning Spanish and French because she wanted to get out of that situation” (participant 6; score: 98.91). “What struck me most was seeing children begging in the streets at the age of three. They were abandoned” (participant 1; score 142.80). This experience also allowed them to learn about other ways of thinking and living (x^2^ = 12.08). “In that house lived Cristina and she was a volunteer. That was her job, and she did not need more. She was much happier than other people who live here with big salaries” (participant 6; score: 105.78). It has also enabled them to reflect (x^2^ = 18.02) on their values. “You were walking around, and you could see a child sleeping in the street, wet and without food. I have realized how much I complain in my life and how much I argue with my mother and sister. That has affected me a lot” (participant 12; score: 166.92). Moreover, they emphasize the change (x^2^ = 5.14) that participating in this activity has meant for their own lives. “You realize that with one week, we are not going to change the lives of the children, we are going to change our lives” (participant 6; score: 119.73).

Class 5: Attitude for Social Transformation

This fifth class is also linked to the categories of social transformation (x^2^ = 18.81, *p* < 0.0001) and well-being (x^2^ = 5.74, *p* 0.01655).

This class, above all, refers to the reflections of students on their lives when they return from experience. Students, upon returning (x^2^ = 58.02) from experience, value how important it is for them to live with basic needs such as health (x^2^ = 25.63), a house (x^2^ = 6.1), and food. “When you come home and see all the food we have at home and the great bed we have, the parents and siblings I have, who care about me and love me very much …” (participant 4; score: 98.62). They also refer to the fact that everything they have learned they have wanted to share with their parents (x^2^ = 20.2) and friends (x^2^ = 29.41). “When I arrive from a trip, I am usually tired, and I always tell my parents: “I will tell you tomorrow,” but this time, when I arrived I wanted to tell everyone about the experience, my father, my mother, my grandfather, my grandmother, and my friends. I wanted to go to their house and tell them everything” (participant 8; score: 197.59). Students think about how they can continue to be committed to those people who are close to them and to society. “I am sure that now I want to do some more volunteering in my city. I want to join some association during this year” (participant 16; score: 79.97). “I am going to smile more and help people, and even if things don’t work out I am going to continue, if I succeed I will celebrate; if my friend can get what she wants, I will celebrate with her. If she cannot, I will help her” (participant 18; score: 80.65).

**Figure 6:**
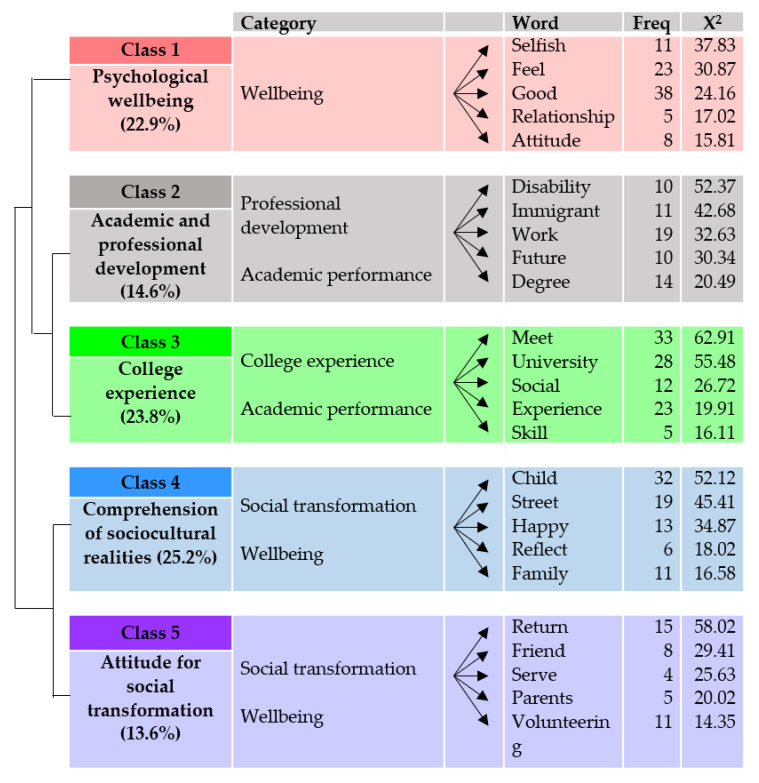
Distribution of classes and their respective units of meaning (post-interviews).

Figure 6 presents the summary of the representation of the main classes, categories, and subclasses resulting from the IRaMuTeQ descending hierarchical classification analysis of the corpus of postinterviews.

## 5. Conclusions

This research aims to explore the role of social interactions in the perceived benefits of students participating in an extracurricular volunteer activity in a developing country. It seeks to explore whether students perceive that these interactions have a positive impact on those dimensions in which the previous literature has identified a significant impact: academic training [6], professional development [8], adaptation to the university context [7], psychological well-being [14], and the development of attitudes of participation in the community [5]. To accomplish this objective, the data collected from the pre- and post-interviews, with the students who participated, were analyzed.

Given the findings from the DHC analysis, the pre-interviews distinguish four classes of functions of the ECA: professional development (class 1); psychological well-being (class 2); professional and academic experience (class 3); and university experience (class 4). These functions are consistent with previous literature. However, in post-interviews five classes are distinguished. Three of them are closely related to the classes taken in the previous phase: psychological well-being (class 1); academic and professional development (class 2); and more enriching university experience (class 3). Additionally, two new classes emerge: understanding sociocultural realities (class 4) and social transformation (class 5).

In line with previous studies [6,8], this study suggests that students, before and after participating in the ECA, perceive that interacting with other students and with people at risk of social exclusion can help them improve their academic and professional practices. Thus, on the one hand, they consider that it can serve to complement the theoretical knowledge they have acquired during the degree and give to that knowledge a more practical and social sense [3]. On the other hand, they believe that it can help them to acquire professional experience and develop skills, such as cooperation and initiative that will favor future professional performance [8]. The volunteers interviewed also corroborate that offering this type of experience where it is possible to interact with peers and with people living in different sociocultural contexts has enriched their university experience [7] and has helped them to reflect on their actions and privileges and to develop a positive attitude toward themselves and others. This, in turn, is linked to obtaining the higher levels of well-being that allow boosting confidence or self-esteem [19,31].

The two additional classes that are obtained from the later interviews, coinciding with the results in other studies [32,34], show the possibility that this voluntary activity offers to understand other social realities and different ways of thinking and to reflect on an individual’s values. Similarly, the participants also value the importance of sharing what they have learned with family and friends and of being committed to close people and the society. Thus, this experience has helped them to become aware of other sociocultural realities and to reflect on possible ways to contribute to the community to increase hope and opportunities for all citizens of the world [4,36]. Therefore, this contribution ranges from continuing to establish quality interactions with family and friends to continuing volunteering activities in other associations.

Regarding the classes that have more presence in the analyzed texts, in the previous interviews, it is the class of psychological well-being and in the interviews carried out after the experience, it is the class of the comprehension of sociocultural realities. The psychological well-being class of the previous interviews is related to feeling better about oneself and to the reflection of the students on their privileges [1]. The class “comprehension of sociocultural realities” of the post phase is linked to the knowledge of social realities and different ways of thinking and the reflection on own values [34]. Therefore, before living the volunteer experience, the class with more presence is very much related to personal development (and that is why words like feel, affect, and selfish are significant), while after living an experience full of interactions, the look of the participants is no longer focused on personal aspects and opens up to other world and social realities (and that is why words like child, street, and family are significant).

The results of this study suggest that promoting volunteer experiences in higher education, in which students have the opportunity to interact with their peers, with other professionals and with people living at risk of social exclusion offers different benefits. Apart from increasing motivation to improve academic and professional performance among the participants, it allows university students to build knowledge shared with others, to develop personal and social skills that contribute to their integral development, and to assume greater commitment and responsibility for their day-to-day actions.

This research work does not aim to generalize the results of the study, which is typical of quantitative studies, but rather to explore an extracurricular volunteer activity to increase the understanding of the phenomenon, specifically of the role of social interactions in the integral development of the participants.

This study provides a detailed overview of the different roles that social interactions provided by ECAs can play in a student’s personal, academic and professional development. Mainly, this study has further strengthened the relevance of participation in ECA to develop skills and competences that promote students’ lifelong learning and the development of a sense of justice and social responsibility. It also opens up new perspectives for the work being done by universities to provide a quality education that facilitates more participatory and meaningful learning, in which the student is the main agent.

To this end, it is necessary to transfer these results to the public institutions that enact laws with curriculum guidelines, to the management teams that manage training processes at the university, and to the teaching staff to raise the awareness of the functions that ECAs perform in an integral development and, consequently, in the training of citizens capable of facing the sustainability challenges of the 21st century.

Despite the relevant practical implications that could be derived from the results obtained in this study, some limitations must be recognized. This work has focused exclusively on the analysis of a specific extracurricular activity; hence, it would be interesting for future research to go deeper into activities of other specific typologies and in universities in different regions or countries.

In conclusion, this study provides scientific evidence that the extracurricular training offered in higher education takes on a significant role during the training process of students. It should, therefore, be considered and included in higher education institutions. The existing literature has shown that social interactions derived from participation in ECAs have a positive impact on the integral formation of students, favoring not only their personal development but also their social development [4].

## Figures and Tables

**Table 1:** Characteristics of the sample. Distribution of the sample by gender, degree, and course studied and previous experience in ECAs.

Gender	Course	Bachelor’s Degree	Previous Experience
Women (*n =* 19) Men (*n =* 4)	1st year (*n =* 4) 2nd year (*n =* 14) 3rd year (*n =* 5)	Study degree in social work or social education (*n =* 10) Study degree in primary education or physical education (*n =* 6) Study degree in law or international relations (*n =* 3) Study degree in psychology (*n =* 2) Study degree in languages or communication (*n =* 2)	Previous experience in volunteer work (*n =* 16) Without previous experience in volunteer work (*n =* 7)

**Table 2:** Question script used in the semi-structured interviews.

No.	Questions	Category
1	How can this experience aid you in changing your attitude?	Personal transformation
2	How do you think this experience will affect your college experience?	College experience
3	To what extent can this experience affect your academic performance?	Academic performance
4	How do you think participating in this experience will make you feel?	Well-being
5	Could you describe how this experience may affect your professional development or the beginning of your professional career?	Professional development
